# INTERGENERATIONAL SILENT DISCO PARTIES FOR OLDER ADULTS LIVING IN LONG-TERM CARE

**DOI:** 10.1093/geroni/igae098.4226

**Published:** 2024-12-31

**Authors:** Ilan Meghelli, Rynnie Cin Ee Son, Julia Banco, Oliver Hang Kin Yu, Shambhavi Arora, Swastika Mondol, Lillian Hung

**Affiliations:** University of British Columbia, Vancouver, British Columbia, Canada; University of British Columbia, Vancouver, British Columbia, Canada; University of British Columbia, Vancouver, British Columbia, Canada; University of British Columbia, Vancouver, British Columbia, Canada; UBC, Vancouver, British Columbia, Canada; University of British Columbia, Vancouver, British Columbia, Canada; University of British Columbia, Vancouver, British Columbia, Canada

## Abstract

Silent disco headphone parties in long-term care (LTC) settings offer a unique way to bring residents together to dance and share musical experiences through wireless, multichannel headphones. Unlike traditional group activities, these parties allow residents to choose their music, creating a personalized yet communal experience. Older adults living in LTC often experience loneliness and social isolation. Silent disco parties can offer a unique opportunity for socialization and enjoyment. This study explored the experiences of 22 older adults in silent disco parties with an intergenerational group of volunteers on the dance floor. Over six weeks, data were collected through video ethnography, including video recordings, conversational interviews, observations, and focus groups with 40 staff members. Thematic analysis identified three themes: intergenerational togetherness, social capacity, and inclusivity. Findings suggest that intergenerational silent disco parties hold promising potential to address loneliness and social isolation in LTC settings. Future research should investigate how these parties can influence residents’ social health and quality of life.

